# Enlightened: addressing circadian and seasonal changes in photoperiod in animal models of bipolar disorder

**DOI:** 10.1038/s41398-021-01494-5

**Published:** 2021-07-05

**Authors:** Richard McCarty, Travis Josephs, Oleg Kovtun, Sandra J. Rosenthal

**Affiliations:** 1 Department of Psychology, Vanderbilt University, Nashville, TN 37240 USA; 2 Neuroscience Program, Vanderbilt University, Nashville, TN 37240 USA; 3 Department of Chemistry, Vanderbilt University, Nashville, TN 37240 USA; 4 Department of Pharmacology, Vanderbilt University, Nashville, TN 37240 USA; 5 Department of Chemical and Biomolecular Engineering, Vanderbilt University, Nashville, TN 37240 USA

**Keywords:** Molecular neuroscience, Human behaviour

## Abstract

Bipolar disorders (BDs) exhibit high heritability and symptoms typically first occur during late adolescence or early adulthood. Affected individuals may experience alternating bouts of mania/hypomania and depression, with euthymic periods of varying lengths interspersed between these extremes of mood. Clinical research studies have consistently demonstrated that BD patients have disturbances in circadian and seasonal rhythms, even when they are free of symptoms. In addition, some BD patients display seasonal patterns in the occurrence of manic/hypomanic and depressive episodes as well as the time of year when symptoms initially occur. Finally, the age of onset of BD symptoms is strongly influenced by the distance one lives from the equator. With few exceptions, animal models useful in the study of BD have not capitalized on these clinical findings regarding seasonal patterns in BD to explore molecular mechanisms associated with the expression of mania- and depression-like behaviors in laboratory animals. In particular, animal models would be especially useful in studying how rates of change in photoperiod that occur during early spring and fall interact with risk genes to increase the occurrence of mania- and depression-like phenotypes, respectively. Another unanswered question relates to the ways in which seasonally relevant changes in photoperiod affect responses to acute and chronic stressors in animal models. Going forward, we suggest ways in which translational research with animal models of BD could be strengthened through carefully controlled manipulations of photoperiod to enhance our understanding of mechanisms underlying seasonal patterns of BD symptoms in humans. In addition, we emphasize the value of incorporating diurnal rodent species as more appropriate animal models to study the effects of seasonal changes in light on symptoms of depression and mania that are characteristic of BD in humans.

Bipolar disorder (BD) is a serious mood disorder that affects approximately 1–3% of the population globally. A defining feature of BD is the recurring manic or hypomanic episodes that may alternate with depressive episodes. Individuals with bipolar I disorder exhibit at least one episode of mania, while those with bipolar II disorder display at least one hypomanic episode and one depressive episode. Hypomania is less severe than mania, although both types of episodes involve significant disturbances to normal functioning, including reductions in sleep, elevated energy levels, restlessness, enhanced self-esteem, rapid generation of new plans and schemes, diminished inhibitions, and increased risk-taking. The age of onset for BD is approximately 20 years of age, with similar rates of occurrence of bipolar I disorder in males and females, but a higher prevalence of bipolar II disorder in females. In addition, the first illness episode tends to occur at an earlier age in bipolar I compared to bipolar II patients. Bipolar I patients display a greater intensity of symptoms but with fewer illness episodes compared to bipolar II patients. Significant levels of disability may occur over the lifespan, and suicide attempts and completions are a significant concern in terms of clinical management [1–4]. In spite of intense research efforts, little is known of the molecular mechanisms underlying BD and there are no established biomarkers to aid in the diagnosis or to gauge treatment effectiveness [5].

## Circadian disturbances in BD

Nearly all people who suffer from mood disorders have significant disruptions in circadian rhythms and the sleep/wake cycle [6]. In fact, altered sleep patterns are one of the major diagnostic criteria for these disorders. Moreover, environmental disruptions to circadian rhythms, including shift work, travel across time zones, and irregular social schedules, tend to precipitate or exacerbate mood-related symptoms of BD [7].

Given the characteristic cycling between manic and depressive episodes that occurs in BD, it is not surprising that clinicians have for many years examined connections between BD and disturbances in circadian and seasonal rhythms. Some of the earliest clinical reports of circadian disturbances in patients with mental disorders were presented by Richter [8,9,], who supplemented clinical case reports from the early 19th century with new examples of patients experiencing two-day cycles of extreme mood swings and disturbances in sleep. Later case reports or clinical studies with small sample sizes noted a lack of normal circadian rhythms in sleep, body temperature, respiration, cardiovascular measures, and water and electrolyte excretion in subgroups of patients with BD [10,11,].

More recent research has shown that disruptions in sleep patterns and circadian rhythms are experienced before the onset of symptoms as well as between and within illness episodes by many individuals diagnosed with BD. These alterations include insomnia, hypersomnia, a reduced need for sleep, increased variability in sleep schedules, lower circadian rhythm amplitude with decreased sensitivity to reductions in sleep, reduced stability of circadian rhythms, and difficulty in adapting to changes in sleep-wake cycles [12–17]. An evening chronotype with a preference for being active at night and a delay in sleep onset are more prevalent in patients with BD than in healthy controls. In addition, BD individuals with a clear-cut evening chronotype exhibited an earlier age of illness onset, more frequent depressive and manic episodes, higher rates of rapid cycling, at least one suicide attempt, and a prior history of psychotic symptoms. Finally, BD patients with an evening chronotype are more likely to attempt suicide and have higher rates of comorbid anxiety and substance use disorders compared to BD patients who do not display an evening chronotype [18–21].

Behavioral manifestations of circadian rhythm disturbances are accompanied by altered temporal patterns of hormone secretion [22]. The rhythmic secretion of melatonin by the pineal gland provides a key signaling molecule that is important in synchronizing biological and behavioral rhythms. Alterations in melatonin secretion are evident in BD patients during as well as between illness episodes, including decreased serum melatonin levels and hypersensitivity of the pineal gland to regulation by ambient light [12]. During manic episodes, daily profiles of circulating melatonin differed between BD patients and healthy controls, with higher daytime levels of melatonin observed in BD patients [23]. In contrast, BD patients with abnormal sleep profiles had lower 24-hour secretion of melatonin compared to BD patients with normal sleep profiles and healthy controls [24].

Cortisol is another hormone that exhibits a circadian rhythm in circulating levels, typically displaying an early morning peak followed by a progressive decline in levels over the remainder of the day. The 24-h cycle of cortisol secretion has been reported to be significantly higher in patients with BD compared to healthy controls [25]. In summary, basic and clinical science data provide overwhelming support for circadian disruptions in endocrine activity and behavior in BD patients, although a clear cause/effect relationship remains to be established.

Chronotherapies, including bright light therapy, sleep deprivation, dark therapy, and sleep phase advance therapy, have been developed to treat underlying circadian disturbances in patients with seasonal affective disorder and non-seasonal major depressive disorder. Of these approaches, bright light therapy has yielded the most promising results and led to a recommendation from the International Society of Bipolar Disorders (ISBD) Task Force on Chronobiology and Chronotherapy for the use of bright light therapy for the acute phase of bipolar depression [13]. Unfortunately, there have been few randomized controlled trials to test the effectiveness of bright light therapy in BD. A recent meta-analysis of 6 randomized controlled trials that examined the effects of bright light therapy versus placebo on bipolar depression failed to detect significant improvements in rates of remission for depressive episodes, improvements in depressive symptom scores, and rates of switching to manic episodes [26]. In contrast, a separate meta-analysis on the efficacy of bright light therapy that included five randomized controlled trials and seven cohort studies reported significant improvements in the severity of depressive symptoms for patients exposed to bright light therapy, especially for light intensities greater than 5000 lx [27].

## Seasonal patterns in BD

Humans retain neurobiological responses to 24-h day-night cycles and seasonal changes in daylength in spite of widespread use of artificial lighting that creates a living environment that is largely independent of dawn-dusk signals associated with sunrise and sunset. Seasonal patterns have been reported in many human functions, from mood to hormone levels to gene expression [28]. Kripke et al. [29] were among the first to suggest that seasonal changes in photoperiod may be involved in the etiology of BD. Since that early report, the results of numerous clinical studies have established that seasonal changes in photoperiod and climatic variables, including ambient temperature and atmospheric pressure, represent critical environmental triggers for alterations in mood in BD [30–34].

Based upon a systematic review of 51 studies published between 1976 and 2013 that examined seasonal patterns of hospital admission rates and the onset of symptoms in BD patients, Geoffroy et al. [33] reported that manic episodes peaked during the spring and summer months, with a minor peak in autumn. In contrast, depressive episodes peaked in early winter and less frequently in summer, with mixed episodes peaking in early spring or summer. Manic episodes and depressive episodes displayed strong seasonal patterns, and depressive episodes were more frequently associated with the bipolar II subtype, comorbid eating disorders, more frequent relapses, and rapid cycling. Finally, women were more likely than men to exhibit seasonal variations in BD [33].

Not all clinical studies have been consistent in finding a seasonal pattern of BD symptoms. Four published reports of prospective studies failed to detect seasonal patterns in BD symptoms, including research conducted in Denmark [35], the United States [36], Canada [37], and six countries in the northern and southern hemispheres [38]. In spite of these negative findings, the preponderance of evidence supports a seasonal pattern of manic/hypomanic and depressive symptoms in a significant percentage of patients with BD [33].

Seasonal changes in duration and intensity of sunlight may also influence the first onset of symptoms related to the BD phenotype. To evaluate this possibility, an international team of investigators led by Dr. Michael Bauer examined the age of onset of BD and the month of symptom onset in 2414 patients with bipolar I disorder from 24 sites in 13 countries that were located 6.3°−63.4° north/south of the equator. They reported that the greater the maximum monthly increase in the amount of solar insolation (the amount of electromagnetic energy from the sun hitting a given location on the earth) at the patient’s location at the onset of illness, the younger the age of onset of BD after controlling for each country’s median age (*P* = 0.006). This resulted in a striking 5-year difference in age of onset of BD symptoms between the locations with the largest (i.e. nearer the poles) versus the smallest (i.e. nearer the equator) monthly increases in solar insolation [39].

A second report from Bauer’s international team of collaborators extended the earlier findings and included data from 4037 bipolar I disorder patients who were living in 318 sites in 43 countries in the northern (74%) and southern (25%) hemispheres during initial BD symptom onset. The results pointed to a significant inverse relationship between maximum monthly increase in solar insolation and age of onset of BD, and this relationship was reduced by about 50% but still remained highly significant in BD patients with no family history of mood disorders [40].

A third report from Bauer’s team investigated seasonal patterns in suicide attempts and completions in 3365 bipolar I disorder patients from 50 sites in 32 countries. Of this sample of BD patients, 1047 (31%) individuals had a history of suicide attempts. They reported a significant inverse relationship between a history of suicide attempts and the ratio of mean winter solar insolation/mean summer solar insolation. This ratio is smallest near the poles where the level of winter solar insolation is greatly reduced compared to the level of summer solar insolation. In contrast, this ratio is largest near the equator where there is relatively little variation in solar insolation over the course of a calendar year. For example, this report noted that the ratio of mean winter solar insolation/mean summer solar insolation was 0.067 for Trondheim, Norway (63.42°N) and 1.056 for Singapore (1.35°N) [41].

In response to the exciting work by Bauer and his colleagues [39–41], Rosenthal et al. [42] analyzed data on monthly levels of solar insolation from 51 locations in the northern and southern hemispheres. Based upon their findings, they suggested that the rate of change in solar insolation and not the absolute monthly amount of sunlight incident on a given area was a key variable that may influence seasonal patterns of manic and depressive symptoms in patients with BD. The rate of change in solar insolation is greatest for sites closer to the poles and least for sites located closer to the equator. Although the studies by Bauer et al. [39–41] and Rosenthal et al. [42] did not establish a causal relationship between seasonal changes in solar insolation and symptoms associated with BD, there is a large body of pre-clinical and clinical research that is consistent with such a relationship [12–14]. Taken together, these exciting findings provide a strong impetus for translational experiments to explore the effects of seasonal changes in photoperiod using animal models relevant for the study of BD.

## Animal models useful for the study of BD

Translational research on animal models of mental disorders plays a critical role in identifying the underlying molecular and circuit-level changes that contribute to a given disorder and points the way to new targets for the development of more effective treatments [43–45]. An additional challenge in developing animal models of BD is to reproduce the spontaneous switch process as individuals with BD progress from one mood state to another mood state of opposite polarity [46,47,]. Finally, we would argue that animal models of BD should reflect the underlying circadian and seasonal disturbances that have been shown to contribute to the etiology of the disorder in humans. In the majority of studies that have employed animal models of BD, investigators have focused on modeling aspects of mania or depression without attempting to replicate the switching process. Only recently have experiments with animal models included manipulations of photoperiod to assess their impact on behavioral and neurobiological markers associated with BD [45].

Several review papers have been published over the past two decades regarding the promise as well as the limitations and challenges associated with animal models of BD [48–57]. A summary of these animal models is presented in Table 1, which includes models of mania as well as models that display a pattern of switching from mania to depression. These models fall into one of several categories:

- *Pharmacological*: single or repeated doses of a drug resulted in significant elevations in locomotor activity (i.e. mania). Some evidence has been presented for cycling between mania and depression based upon repeated administration of amphetamine or cocaine.
- *Environmental*: sleep deprivation resulted in increased levels of locomotor activity, a sign of mania.
- *Genetically selected strains*: two inbred strains of mice, Madison mice and Black Swiss mice from Taconic, are hyperactive while the Hyperactive (HYPER) line of rats displays evidence of stress-induced bursts of activity followed by prolonged period of depression-like behavior.
- *Genetically modified strains*: these animal models of BD have capitalized on the manipulation of known or suspected risk genes for BD and include knock-outs, overexpression models, and models produced by in vivo mutagenesis. This approach has been by far the favored path for developing animal models of BD over the past two decades (Table 1).

**Table 1:** A summary of key characteristics and behavioral phenotypes of various animal models of BD.

Animal model	Key features	Behavioral characteristics of model	BD patterns	References
*Pharmacological*				
Amphetamine (AMPH)	Acute/chronic peripheral administration	↑ locomotor activity related to DA, some evidence for cycling from depression	Mania + cycling (?)	[88–95]
Cocaine	Chronic peripheral administration	↑ locomotor activity related to DA, time-dependent sensitization	Mania + cycling (?)	[96]
Fenproporex	Converted to AMPH in vivo, acute and chronic peripheral administration	↑ locomotor activity	Mania	[97]
GBR 12909, an inhibitor of the DA transporter (DAT)	Acute peripheral administration	↑ activity and perseverative patterns of locomotion for several hours	Mania	[98]
Ouabain	Acute icv administration	↑ activity, ↓ reversal learning	Mania	[99–101]
*Environmental*				
Sleep deprivation	Sleep deprivation (SD) using the platform method with testing afterwards, vary duration and frequency of SD	After sleep deprivation: ↑ activity, insomnia, ↑ irritability, ↑ aggression, ↑ sexual behavior, evidence of sensitization to repeated SD	Mania	[102,103,]
*Genetically selected strains*				
Black Swiss mice	Mania-like phenotype observed in mice from Taconic Biosciences but not Charles River Labs	Hyperactive, ↓ immobility in FST, ↑ response to AMPH, ↑ aggression, ↑ saccharin preference	Mania	[104]
Hyperactive (HYPER) rat	Derived from a single litter of Sprague–Dawley rats that were discovered to be hyperactive at night.	Bursts of activity following exposure to a stressor followed by prolonged period of depression-like behavior, ↑ anxiety, anhedonia, ↑ alcohol intake.	Mania → depression	[105]
Madison (MSN) mice	Developed at Univ. of Wisconsin for exercise physiology experiments, mice display a mania-like phenotype	Hyperactive day and night, ↓ immobility in FST, ↓ daytime sleep, sensitive to changes in photoperiod	Mania	[76,106,]
*Genetically modified strains*				
DNA polymerase subunit gamma (POLG) transgenic mice (Tg+/−)	POLG is the catalytic subunit of mitochondrial (mt) DNA polymerase, POLG mutation was limited to brain neurons	Tg mice displayed a distortion in the daily circadian activity pattern, ↑ activity associated with estrous, antidepressant-induced mania	Mania	[107]
Myshkin mice (Myk^+/−^)	In vivo mutagenesis-derived strain, point mutation in Na+,K+-ATPase α3	↑ exploration of a novel object, ↑ open field activity and ↓ habituation, ↓ sleep, ↓ anxiety-like behaviors, ↑ sucrose pref.	Mania	[108,109,]
*Clock* mutant mice	Identified through in vivo mutagenesis as a deletion of exon 19 that affects circadian rhythms	↓ anxiety, ↑ locomotion, ↓ immobility in FST, ↑ sucrose pref., ↑ response to cocaine, ↓ sleep, mood cycling from daytime mania → night time euthymia	Mania → euthymia	[110–113]
D-box binding protein mice (DBP−/−)	DBP is a transcription factor that regulates the period length of the cellular circadian clock	↑ response to AMPH, ↑ activity after 4 weeks of chronic stress, ↑ activity after sleep deprivation	Depression → mania	[114,115,]
Extracellular regulated kinase 1 (ERK1) KO mice	ERK pathway is a major signaling cascade mediating the effects of BDNF	↑ activity, ↑ reward seeking, ↓ immobility in FST, ↑ response to AMPH	Mania	[116]
Histidine triad nucleotide binding protein 1 (HINT1-/-) mice	Identified as a risk gene for BD by GWAS, regulates activities of GPCRs and NMDA receptors in brain	↑ activity, ↓ anxiety, ↑ aggression, ↑ sucrose pref., ↓ immobility in FST, ↑response to apomorphine or AMPH, switch from mania → depression after acute stress	Mania → depression	[117,118,]
SH3 and multiple ankyrin repeat domains 2 (Shank2) mice	Shank2 mice were generated by deletion of exon 24 (Δe24−/−) or by targeted deletion in the forebrain	↑ activity in home cage and OF, lack of habituation of OF activity, ↑ response to AMPH, ↓preference for sucrose, ↑ reward-seeking behavior in an operant task, fragmentation of activity under constant darkness, abnormal social behavior, ↓ spatial learning	Mania	[119]
SH3 and multiple ankyrin repeat domains 3 (Shank3) Tg mice overexpressing Shank3	Shank proteins are multidomain scaffold proteins of the postsynaptic density	Shank3 overexpression leads to ↑ locomotor activity and ↑ locomotor response to AMPH, seizures.	Mania	[120]
Ankyrin 3 (ANK3^+/−^) mice ANK3 cKO mice	ANK3 codes for Ankyrin-G, a large scaffold protein involved in formation and maintenance of axon initial segment, identified as a risk gene for BD and SCZ by GWAS	↓ anxiety, ↑ activity during daytime, ↑ sucrose pref., switch from mania → depression after isolation housing or CSDS, reversion to a mania-like state 7 days after the end of CSDS	Mania → depression → mania	[121,122,]
Glycogen synthase kinase 3β (GSK-3β)	GSK-3β is involved in several signaling pathways coupled to neuronal receptors	Overexpression of GSK-3β results in a mania-like phenotype with ↑ activity, ↓ habituation, ↑ acoustic startle response	Mania	[123]
GluA1 KO mice	GluA1 is an ionotropic AMPA receptor subunit associated with BD in human studies.	↑ activity and exploration in a novel cage and OF, ↓ anxiety in EPM, ↓ immobility in FST and TST, ↑ sucrose preference, ↑ response to novelty	Mania	[124]
Glutamate receptor 6 (GluR6) KO mice	GluR6 is one of the kainate receptors and has been shown to be associated with BD in human studies.	↑ OF and home cage activity, lack of habituation to repeated testing, ↑ response to AMPH, ↑ aggression, ↓ anxiety, ↑ risk taking, ↓ immobility in FST	Mania	[125]
B-cell lymphoma-2 (Bcl-2^+/−^) mice	Modulator of cell growth and survival in brain, inhibits programmed cell death	↑ reward seeking, ↑ sensitivity to AMPH, vulnerable to mania or depression	Vulnerable to mania or depression	[126]
L-type voltage-gated Ca^++^ channel Cav1.2 isoform, α-1C subunit (CACNA1C) mice (Cacna1c+/−)	Cav1.2 accounts for 85% of L-type channels in mouse brain, SNPs of CACNA1C associated with BD by GWAS	Sex-specific behavioral changes that suggested a mood-stabilized phenotype modified by sex for some but not all behaviors.	Unstable mood	[127]
Early growth response gene 3 (Erg3−/−) KO mice	Erg3 is an immediate early gene that is regulated in part by BDNF, ↓levels in PFC of post mortem BD brain, involved in synaptic plasticity and cognition	Erg3^-/-^ KO mice display ↑ behavioral and HPA axis responses to mild stress, ↑ OF activity, ↓ learning, ↓ habituation to social cues, ↑ aggression, ↑ impulsivity	Mania	[128–130]
Dopamine transporter (DAT) KD mice or DAT+/− mice	DAT plays a critical role in the reuptake of DA from the synaptic space back into DA nerve terminals, DAT KD mice have a 90% reduction in DAT expression	↑ activity in OF, ↑ risk taking behavior, ↑ reward seeking, ↑ reversal learning, behavioral profile similar to BD patients in the Behavioral Pattern Monitor, evidence of cycling based upon photoperiod changes	Mania → Depression dependent on changes in photoperiod	[77,131–137]
Orthodenticle homeobox 2 (Otx2)	Otx2 is a transcription factor that plays a key role in the development of brain DA and 5-HT neurons. Studies were conducted in mice overexpressing Otx2 (En1+/Otx2)	↑ DA neurons, ↓ 5-HT neurons, En1 + /Otx2 mice displayed prolonged periods of increased or decreased locomotor activity as well as risk taking, habituation, and hedonic behaviors. Evidence of spontaneous mood cycling?	Spontaneous mood cycling (?)	[138]
VTA GABAergic neurons	VTA GABAergic neurons were lesioned or inhibited chemogenetically (VTA^Vgat^) in mice.	Wakefulness with evidence of mania, ↑ activity, ↑ response to AMPH, ↓ immobility in FST and TST, ↑ sucrose preference, impaired sleep homeostasis.	Mania	[139]
Neurotrophic Receptor Tyrosine Kinase 1 (NTRK1)	Codes for the NGF receptor, important for the development of cholinergic neurons, identified as a BD risk gene in a multi-generational family.	A conditional knock-in mouse line was developed that expressed the mutation in brain, affected mice may serve as a model of bipolar depression linked to cholinergic signaling.	Depression	[140]
REV-ERBα knockout mice	REV-ERBα is a circadian nuclear receptor that has been shown to be associated with BD by GWAS.	REV-ERBα KO mice led to increased brain DA activity, ↓ immobility in FST and TST, ↓ anxiety in EPM, ↓ habituation to OF testing, ↑ aggression, ↑ fear responses, altered DA signaling was evident	Mania	[141]
ErbB4 deletion in LC	ErbB4 is a receptor tyrosine kinase that is involved in neuronal excitability and synaptic plasticity, expressed in LC NE neurons, coding variants of ErbB4 associated with BD in humans.	↑ OF activity, ↓ anxiety in EPM, ↓ immobility in FST, ↑ sucrose preference, ↑ in activity of DA and NE neurons associated with mania-like behaviors.	Mania	[142]
Phospholipase Cγ1 targeted deletion in the forebrain (PLCG1^f/f^)	PLCG1 is involved in second messenger generation in response to multiple neurotransmitters and neuromodulators, also associated with BD in human studies.	↑ activity, ↓ anxiety in OF and EPM, ↓ immobility in FST, ↑ sucrose preference, ↓ fear memory, ↑ startle response, stressors did not result in switch from mania → depression.	Mania	[143]

Also specified for each animal model is whether it captures aspects of mania and/or depression and if there is a tendency for animals to cycle between the two extremes of mood. Selected references for each model are also included.

*AMPH* amphetamine, *CSDS* chronic social defeat stress, *DA* dopamine, *EPM* elevated plus maze, *FST* forced swim test, *5-HT* serotonin, *icv* intracerebroventricular, *KD* knock down, *KO* knockout, *NE* norepinephrine, *OF* open field, *TST* tail suspension test

Given the plethora of animal models of BD, how does one gauge the appropriateness of a given animal model against what is known about the disorder in humans? Early efforts by McKinney [58,59,] and Willner [60] sought to establish criteria for evaluating the utility of a given animal model for enhancing our understanding of mental disorders in humans, with a particular emphasis on animal models of major depressive disorder. Building upon these and other earlier efforts, Belzung and Lemoine [61] have developed a multifactorial framework for assessing the value of animal models of mood disorders. Their framework included five categories of validity relevant for assessing the benefits of utilizing various animal models of mental disorders (Table 2). With respect to pathogenic and mechanistic validity, the animal models of BD presented in Table 1 have largely failed to include seasonal changes in photoperiod in spite of the fact that this is a well-established triggering factor that contributes to the onset of symptoms in individuals at risk of BD. In addition, all of these animal models are based upon nocturnal mice or rats and this presents additional challenges as will be described below.

**Table 2:** Major categories of validity relevant to the assessment of animal models of BD.

Type of Validity	Description
Homological validity	Appropriateness of the species and strain of the animal model to study aspect(s) of the human mental disorder.
Pathogenic validity	Tendency of the animal model to display pathological processes that resemble those of the human disorder. These pathological processes include early environmental factors that render an organism vulnerable to develop the disorder later in life and triggering factors that impact a vulnerable or a control organism in adulthood and lead to the disorder.
Mechanistic validity	Similarity of the mechanism of disease in the animal model to the actual or hypothesized mechanism underlying the onset/maintenance of the human disorder. The mechanism may be behavioral or neurobiological in nature.
Face validity	Consistency of observations between the animal model and the human disorder, including behaviors and biomarkers.
Predictive validity	Effectiveness of the animal model in mirroring the etiology of disease onset and the response to a therapeutic agent (drug or other treatment) in the human disorder.

These categories and their descriptions were adapted from Belzung and Lemoine [61].

## Seasonal changes in photoperiod in animal models of BD

Our focus in this review is to highlight the need for more sophisticated approaches to controlling the photoperiod of laboratory mice and rats used to model BD to permit a robust analysis of circadian and seasonal effects of light on BD symptoms. Ideally, laboratory studies using animal models should come as close as possible to approximating conditions for disease onset and progression in humans. It is surprising, therefore, that so few studies using animal models of BD have explored the impact of seasonal changes in photoperiod on symptom onset given our ability to manipulate photoperiod with great precision in vivarium rooms.

**Fig. 1:**
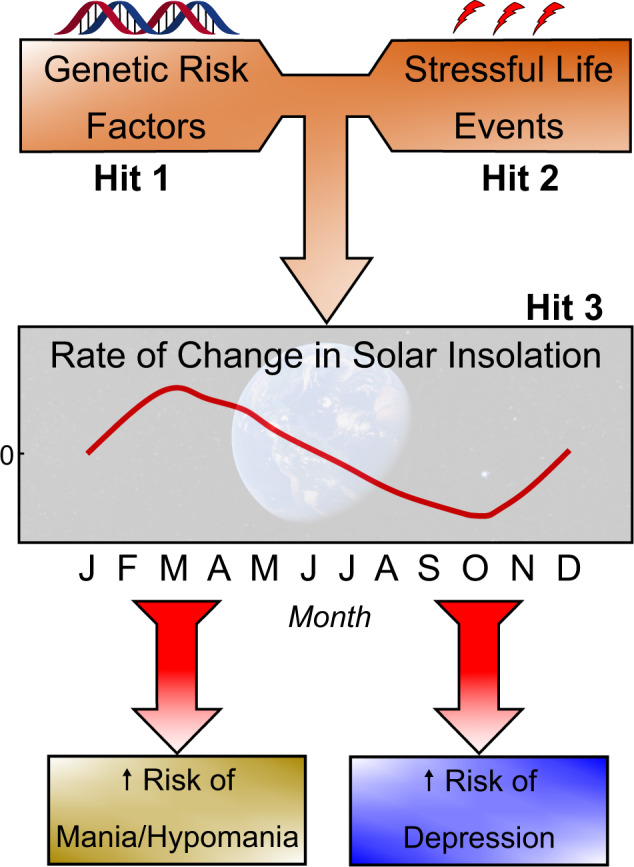
A 3-hit model of BD involving an interaction of risk genes (Hit 1) and life stressors (Hit 2) that is filtered through seasonal changes in photoperiod (Hit 3), represented as rates of change in solar insolation. An idealized pattern of rates of seasonal changes in photoperiod for a location north of the equator is presented, and details relating to this measure may be found in Rosenthal et al. [42]. This model is a modification of the 3-hit model of vulnerability and resilience as originally proposed by Daskalakis and colleagues [62] and is based in part on the social zeitgeber theory of affective disorders [63,64,].

To situate our review in a theoretical context, we have modified the 3-hit concept of vulnerability and resilience to stress-related mental disorders originally proposed by Daskalakis and colleagues [62], but with several key alterations (see Fig. 1). We suggest that risk genes for BD (Hit 1) interact with stressful life experiences in late adolescence and/or early adulthood (Hit 2), and this interaction is then filtered through a lens of circadian and seasonal changes in photoperiod (Hit 3) to alter the risk of occurrence of symptoms of mania/hypomania or depression (Fig. 1). Our model also draws substantially on the social zeitgeber theory of depressive episodes as originally proposed by Ehlers et al. [63] and later extended by Grandin et al. [64] to (hypo)manic episodes.

In most experiments with animal models useful for the study of BD to date, laboratory animals experience an instantaneous onset of light followed later in the day by an abrupt switch to total darkness, with the photoperiod remaining unchanged for the duration of most experiments. Few investigators have attempted to program a gradual onset of light in the morning (dawn) and a gradual offset of light in the evening (dusk), even though digital light control systems are available with such capabilities. In addition, the standard photoperiod reported for many studies of laboratory mice and rats is set at a 12:12 light–dark photoperiod, which itself is artificial. Areas away from the equator experience equal amounts of light and darkness in a single day for only a brief transitional period during the increasing daylengths of spring and the decreasing daylengths of fall. In fact, Metzger et al. [65] noted that a 12:12 light–dark photoperiod corresponds to times of the year when laboratory mice housed in semi-natural outdoor enclosures switched back and forth from unstable winter chronotypes to more stable summer chronotypes and vice versa. Given these findings, these investigators expressed concern for employing a 12:12 light–dark photoperiod as the default setting for laboratory studies of mice and rats.

The experiments conducted by Metzger and colleagues [65] have important implications for researchers interested in developing or refining currently utilized animal models of BD. They studied patterns of locomotor activity over the course of a calendar year in C3H and C57BL/6 J inbred mice that were housed singly in semi-natural outdoor enclosures that were provided with nest boxes, nesting material, and ad lib quantities of food and water. The experiments were conducted in Frankfurt am Main, Germany at 50°N latitude. The enclosures provided protection from predators, but the mice experienced the full range of changes in ambient light (natural sunlight as well as street lighting at night) and temperature as well as human activity over the period of study. Activity levels were quantified by an infrared locomotion detection system rather than by running wheels, which are more commonly employed in laboratory studies. The results were quite striking. C3H mice, which display a robust circadian pattern of melatonin secretion from the pineal gland, exhibited an earlier chronotype and a monophasic activity pattern whereas C57BL/6J mice displayed a later chronotype and a biphasic activity pattern. It should be noted that C57BL/6J mice lack the ability to synthesize a key enzyme required for melatonin biosynthesis [66]. Activity patterns for mice of both strains were predominantly nocturnal. In summer months with shorter nights and warmer temperatures, C3H mice displayed brief bouts of activity but with high levels of activity per bout. During the winter months with longer nights and colder temperatures, C3H mice had longer bouts of activity but with lower levels of activity per bout. These seasonal differences in lengths of activity bouts and total amount of activity were not observed in C57BL/6J mice [65]. The absence of seasonal changes in plasma levels of melatonin in C57BL/6 mice would be expected to influence the sleep-wake cycle as well as measures of mood-related behaviors based upon previous findings in laboratory studies of mice and clinical studies of patients with affective disorders [67].

Compared to ambient temperature, relative humidity, barometric pressure, and other climatic variables, seasonal changes in photoperiod provide the most dependable environmental cue for synchronizing circannual cycles of physiological and behavioral adaptations in humans and other animals [68]. For example, humans living at higher latitudes in Europe experience dramatic seasonal changes in photoperiod that are accompanied by significant changes in immune function, with a proinflammatory state evident in winter months [69].

Studies of seasonally breeding birds and mammals have revealed that the pars tuberalis (PT) is a critical site for regulating seasonal changes in physiology and behavior. The PT, a part of the anterior lobe of the pituitary gland, wraps around the infundibular stalk and extends up to the median eminence. PT-specific thyrotrophs express a high density of MT1 receptors that bind melatonin and this enrichment of MT1 receptors has been observed in a range of mammals, including seasonal and non-seasonal breeders. The PT is ideally positioned between the basal hypothalamus and the pituitary and is in direct contact with the median eminence to influence seasonal patterns of behavior through anterograde (toward the anterior pituitary) and retrograde (toward the hypothalamus) signaling pathways. PT-specific thyrotrophs synthesize and release the β subunit of thyrotropin releasing hormone (TSHβ), which can be retrogradely transported to the hypothalamus and bind to TSH receptors on β2 tanycytes, specialized ependymal cells that line the base of the third ventricle [70]. Activation of TSH receptors on tanycytes leads to increased expression of deiodinases, which catalyze the conversion of thyroxine (T4) to the biologically active form, triiodothyronine (T3) [71–73].

**Fig. 2:**
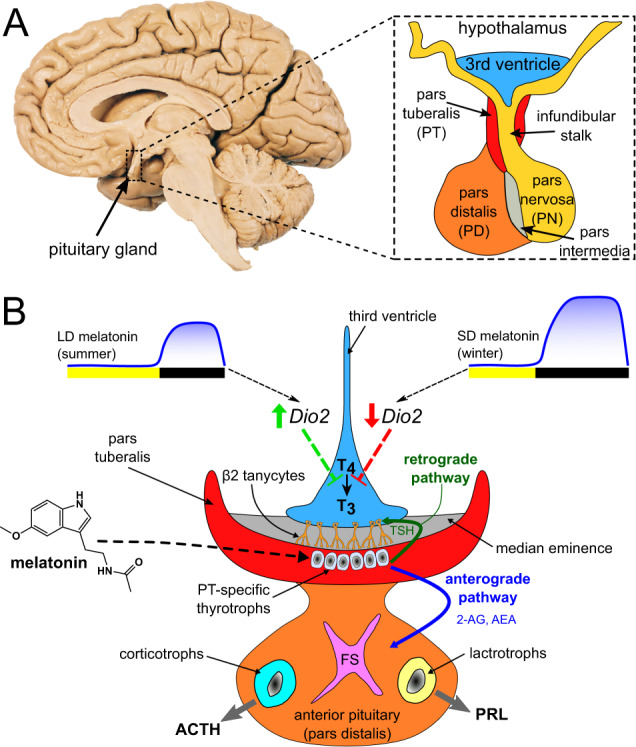
Overview of the possible role of the pars tuberalis (PT) in seasonal variations in mood and energy levels that are characteristic of BD. **A** A mid-sagittal section of the human brain, with an expanded diagram of the basal portion of the hypothalamus and third ventricle (V3) and the infundibular stalk that connects the pituitary gland to the hypothalamus. **B** Circulating levels of melatonin at night are reduced during the long days (LD) of summer and increase with the short days (SD) of winter. This diagram depicts a coronal section through the V3, PT, and the anterior pituitary. The PT wraps around the infundibular stalk and contains a high density of melatonin (MT1) receptors on PT-specific thyrotrophs. A retrograde pathway from the PT involves the release of thyroid stimulating hormone (TSHβ) that stimulates receptors on β2 tanycytes that line the base of V3. Under long-day conditions of summer, type 2 deiodinase is upregulated to facilitate the conversion of the prohormone thyroxine (T4) to the bioactive triiodothyronine (T3). T3 is taken up into brain areas to increase reproductive functions, energy levels and mood. PT-specific thyrotrophs also communicate through an anterograde pathway to the pars distalis (anterior pituitary) by releasing the endocannabinoids, N-arachidonoylethanolamine (anandamide, AEA) and 2-arachidonoylglycerol (2-AG), which stimulate folliculo-stellate (FS) cells in the anterior pituitary, leading to the release of ACTH from corticotrophs and prolactin from lactrotrophs [70–74].

A current model of photoperiodic entrainment posits that changes in the circulating melatonin signal are transduced by a circadian-based “coincidence timer” in the PT. This coincidence timer quantifies the duration of the melatonin signal to dictate the amplitude of expression of the transcriptional co‐activator, *eyes absent homolog 3* (*Eya3*), which regulates *Tshß* gene expression. In long-day photoperiods of summer, increased expression of *Eya3* leads to up‐regulation of *Tshß*, whereas this system is down-regulated in short-day photoperiods of winter [74] (Fig. 2). Our current working hypothesis is that increased availability of T3 modulates energy-, reproduction-, and mood-related neural circuits relevant for the expression of BD symptoms. Another possibility for light to affect mood-related circuits and behaviors is through intrinsic photoreceptive retinal ganglion cells that project to the perihabenular nucleus of the thalamus, with downstream influences on areas of prefrontal cortex [75].

## Modeling seasonal changes in photoperiod

We conducted a review of 94 published empirical articles encompassing the most relevant animal models of BD, many of which are cited in this paper (refer to Table 1). Our review revealed that 9 articles did not specify the photoperiod employed in the experiments. Of the remaining 85 articles, 81 (95.3%) utilized a 12:12 h light–dark photoperiod, one utilized a 14:10 h light–dark photoperiod, and one utilized a 10:14 h light–dark photoperiod. Two other studies included manipulations of photoperiod to study seasonal changes, but utilized a 12:12 h light–dark photoperiod as the default photoperiod and a basis for comparison to results from the long-day and short-day photoperiods (see below). In only a few instances have investigators systematically manipulated photoperiod to reproduce in an animal model the seasonal changes in mood that have been described for humans with BD. Two examples are noteworthy and are summarized below.

Gammie and his colleagues [76] examined the effects of different seasonally relevant photoperiods on the mania-like phenotype of Madison (MSN) mice. MSN and ICR outbred control mice were housed in 1 of 3 photoperiods from weaning at 3 weeks of age until 12–13 weeks of age: 6:18 h light-dark photoperiod, 12:12 h light-dark photoperiod, or 18:6 h light-dark photoperiod. Recordings of 24 h activity profiles revealed that MSN mice had the greatest levels of locomotor activity during long days (18:6 h light-dark photoperiod) and were more active during the light–dark transitions compared to the other two photoperiods. Consistent with their mania-like phenotype, MSN mice were more active when housed in each of the three photoperiods compared to ISR mice. These investigators concluded that MSN mice have a mania-like behavioral pattern with a comorbid seasonal component.

Young and colleagues [77] examined seasonal extremes of light–dark cycles on behavioral and neural measures using wild-type (WT) mice and mice heterozygous for the dopamine (DA) transporter gene (DAT-HZ), both of which were derived on a C57BL/6J background. DAT-HZ mice expressed 50% of WT levels of DAT protein in brain areas. Mice were housed for at least 2 weeks in one of three photoperiods: long-active (LA, 5:19 h light–dark photoperiod), short-active (SA, 19:5 h light–dark photoperiod), or control (NA, 12:12 h light–dark photoperiod). The time of lights-on for the 3 photoperiods was adjusted such that each group shared a 5-hour period of darkness each day when behavioral testing occurred. DAT-HZ mice housed in a SA photoperiod exhibited increased immobility in the forced swim test, a depression-relevant behavioral measure. In the elevated plus maze, DAT-HZ mice housed in an LA photoperiod displayed more frequent open arm entries during elevated plus maze testing, a mania-relevant behavioral measure. In addition, DAT-HZ mice in LA conditions were more sensitive to rewards, while in SA conditions they were more sensitive to punishment and loss in two learning tasks. Photoperiod also affected the balance between tyrosine hydroxylase (TH)-positive and somatostatin-positive neurons in the hypothalamic paraventricular nucleus (PVN). Compared to WT mice housed in the NA photoperiod, DAT-HZ mice had reductions in TH-positive neurons but increases in somatostatin-positive neurons when housed in the SA photoperiod. The opposite was true for the LA photoperiod, with increases in TH-positive neurons and decreases in somatostatin-positive neurons. These changes in neurotransmitter phenotype may underlie the switching process in this animal model of BD [77,78,].

Several concerns have been raised regarding the experimental design described above for the study from Young’s laboratory [79]. C57BL/6J mice lack the ability to synthesize melatonin due to a naturally occurring point mutation in the gene coding for serotonin N-acetyltransferase [66]. In addition, these investigators flipped the designations for typical winter and summer photoperiods, such that for them, a short-day cycle (5:19 h light-dark photoperiod) was labeled as “summer-like” [long-active (LA) photoperiod] while a long-day cycle (19:5 h light-dark photoperiod) was labeled as “winter-like” [short-active (SA) photoperiod]. Increasing the number of hours of darkness in a 24-h period does not necessarily translate into increases in total daily activity in a nocturnal species such as laboratory mice as noted by Metzger et al. [65]. These concerns call for a significant re-interpretation of these intriguing results. In spite of these concerns, however, this report and related ones [77,80, 81] represent a promising starting point for testing the effects of seasonal changes in photoperiod on mania-like and depression-like phenotypes.

## Diurnal versus nocturnal: that is the question

Perhaps the greatest challenge facing researchers interested in developing valid animal models of bipolar disorder is the mismatch between diurnal humans and nocturnal laboratory mice and rats. As is evident from the information presented in Table 1, experiments on animal models of BD have been conducted exclusively using nocturnal strains of laboratory mice and rats. Although the findings from these experiments have provided valuable information on the regulation of mania- and depression-like behaviors and cycling between mood states, some fundamental differences between laboratory mice with nocturnal rhythms and humans with diurnal rhythms remain. A simple example captures these differences: bright light inhibits locomotor activity and promotes sleep in nocturnal rodents while it increases locomotor activity and levels of arousal in diurnal rodents and humans.

Several species of diurnal rodents have been studied as animal models of depression and seasonal affective disorder [82]. These diurnal rodent species include degu (*Octodon degus*), golden spiny mice (*Acomys russatus*), fat sand rats (*Psammomys obesus*), Nile grass rats (*Arvicanthis niloticus*), and Mongolian gerbils (*Meriones unquiculatus*). It is apparent from these initial studies that diurnal rodent species respond to manipulations of photoperiod in a manner similar to humans and often distinct from nocturnal rodent species. For example, housing diurnal rodents but not nocturnal rodents under a short-day photoperiod (5:19 h light-dark photoperiod) resulted in the development of a depression- and anxiety-like phenotype [82]. In contrast, nocturnal and diurnal rodent species respond in a similar fashion when exposed to dim light at night with decreases in learning and memory and increases in depression-like behaviors [83].

From these and related studies, it has become clear that the brains of diurnal rodents are not simply a phase-reversed version of the brains of nocturnal rodents. Experiments with diurnal rodents have the potential to illuminate circadian and seasonal effects of light on mood-related behaviors and brain circuits in humans [84,85,]. Although diurnal rodents have been utilized as animal models for seasonal affective disorder and major depressive disorder [86], we are unaware of any reports of diurnal rodents being utilized as animal models of BD. This represents a new and exciting opportunity to refine and extend previous experiments by employing diurnal species to more closely match humans with BD.

From the important study by Metzger and colleagues [65], it is apparent that C3H mice demonstrated robust seasonal rhythms in activity when housed for 1 year in an outdoor enclosure. The same is probably true for other inbred and outbred strains of laboratory mice that exhibit significant daily and seasonal rhythms in melatonin secretion. These results can inform the ways in which investigators attempt to model the effects of seasonal changes in photoperiod on shifts in mood-related behaviors in animal models of BD. We are unaware of any experiments that have been conducted to date where an animal model of BD experiences seasonally relevant changes in daylength to simulate the natural changes in photoperiod that occur over the course of a year. Our previous work suggested that the rate of change in solar insolation was a critical variable in promoting the onset of symptoms of mania/hypomania in the spring and symptoms of depression in the fall [42]. This hypothesis awaits careful experimental confirmation in the laboratory. A new opportunity to employ diurnal rodents as potential animal models of BD was presented and would address some of the critical shortcomings in using nocturnal strains of laboratory mice and rats to model aspects of BD [82–86]. Such an opportunity does come with significant costs, however, as many foundational experiments would be required to demonstrate the advantages of diurnal species to current models that utilize nocturnal species. A major unknown is whether any diurnal species would display a tendency to cycle between depression- and mania-like phenotypes under specific seasonally relevant photoperiods.

## Future directions

Listed below are some of the many important issues to be considered in future experiments involving animal models of BD:

1. Program vivarium light controls to more closely mimic the changes in natural light intensity over the course of a 24-h period, with a ramp-up of light intensity in the morning (dawn) and a ramp-down of light intensity in the evening (dusk).
2. Consider using a 13:11 h light-dark photoperiod as the default photoperiod to avoid the unstable nature of the transitional 12:12 h light-dark photoperiod as noted above.
3. Avoid the use of C57BL/6 strains of mice in laboratory experiments that include manipulations of photoperiod because these mice lack the ability to synthesize melatonin. This is an unfortunate situation given that many of the genetic manipulations that have been important in the study of mood-related circuits and behavior in laboratory mice have been conducted in animals using a C57BL/6 background.
4. Explore the impact of rates of change in light intensity comparable to the patterns occurring in the spring and fall months on molecular and circuit-level changes in the brain and on mania- and depression-like behaviors in adolescence and adulthood in animal models of BD. Actual data for solar insolation at various locations around the globe could be employed to guide parameters for manipulating rates of change in light intensity.
5. Explore individual differences in response to seasonally relevant changes in photoperiod in laboratory animals to determine why some BD patients display seasonal patterns and others do not. It is especially critical to include females as well as males in all such studies given the sex differences in BD symptoms.
6. Assess the influence of seasonally relevant changes in photoperiod on responses to acute and chronic stressors in adolescence and adulthood in animal models of BD.
7. Address the effects of seasonally relevant changes in photoperiod on the switch process from a mania-like behavioral phenotype to a depression-like phenotype and vice versa in animal models of BD.
8. Investigate the benefits of employing a diurnal rodent species as a new animal model of BD. There may be distinct benefits to using a diurnal species versus nocturnal strains of laboratory mice and rats as has been the convention for many decades.

In this review, we have highlighted some of the opportunities to explore more deeply the ways in which risk genes interact with stressful stimuli against a backdrop of seasonal changes in photoperiod to influence the expression of behavioral changes associated with BD. Considering that the current edition of the *Diagnostic and Statistical Manual* [87] has adopted a seasonal pattern specifier for BD I and BD II, it is surprising that most studies to date with animal models of BD have not taken this critical variable into account.

The 3-hit model we propose here fits well with the information presented in this review. The research opportunities we have described may lead to the identification of novel molecular targets for the treatment of BD by systematically varying the seasonally relevant photoperiod when experiments are conducted. A careful consideration of the potential contributions of diurnal animal models of BD also seems warranted.
