# Correction: Enlightened: addressing circadian and seasonal changes in photoperiod in animal models of bipolar disorder

**DOI:** 10.1038/s41398-021-01521-5

**Published:** 2021-07-21

**Authors:** Richard McCarty, Travis Josephs, Oleg Kovtun, Sandra J. Rosenthal

**Affiliations:** 1 Department of Psychology, Vanderbilt University, Nashville, TN 37240 USA; 2 Neuroscience Program, Vanderbilt University, Nashville, TN 37240 USA; 3 Department of Chemistry, Vanderbilt University, Nashville, TN 37240 USA; 4 Department of Pharmacology, Vanderbilt University, Nashville, TN 37240 USA; 5 Department of Chemical and Biomolecular Engineering, Vanderbilt University, Nashville, TN 37240 USA

**Keywords:** Molecular neuroscience, Human behaviour

Correction to: *Translational Psychiatry* 10.1038/s41398-021-01494-5, published online 05 July 2021

The original version of this article unfortunately contained a mistake in Table 1. All blank boxes (☐) in Table 1 columns 3 and 4 should be replaced with the horizontal solid arrow (→). The remaining dashed horizontal arrow (-->) in the bottom cell of column 3 of Table 1 should be replaced with the horizontal solid arrow (→) as well. We apologize for this error caused by the typesetter. The original article has been corrected.

